# 1636. Prevention of respiratory syncytial virus hospitalizations (RSVH) in US infants who paid through Medicaid would have a disproportionate benefit for Black, Hispanic, Asian/Pacific Islander, Native American and Other race/ethnicity infants, and have a substantial impact on overall RSVH burden and related costs: Rates of respiratory syncytial virus hospitalization (RSVH) among US infants by race/ethnicity and insurance payer, NIS 2018-2020

**DOI:** 10.1093/ofid/ofad500.1470

**Published:** 2023-11-27

**Authors:** Christopher B Nelson, Xiaohui Jiang, Mina Suh, Jon Fryzek

**Affiliations:** Sanofi, Swiftwater, Pennsylvania; EpidStrategies, A Division of ToxStrategies, Inc., Rockville, MD; Epidstrategies, Mission Viejo, California; EpidStrategies, A Division of ToxStrategies, Inc., Rockville, MD

## Abstract

**Background:**

Respiratory syncytial virus (RSV) is the leading cause of US infant hospitalization [1]. The objective of this work is to describe the rate of US infant RSV hospitalization (RSVH) and related charges by race/ethnicity and type of insurance payer.

**Methods:**

Using the US national Healthcare Cost and Utilization Project (HCUP) National Inpatient Sample (NIS) for 2018-2020, we identified US infant RSVH and charges by RSV diagnosis in any position, race and Hispanic origin of the mother (race/ethnicity), and type of insurance payer.

**Results:**

RSVH Rates and Burden: Rates of US infant RSVH are highest among Native American infants, are similar for White, Black, and Hispanic infants, and are lower among Other/Unknown and Asian/Pacific Islander infants. The trend of RSVH rates being higher among infants covered by Medicaid is consistent across each race/ethnicity group (**Figure 1**). This results in a greater proportion of the US infant RSVH burden among Black, Hispanic, Asian/Pacific Islander, Native American, and Other/Unknown infants being among infants covered by Medicaid (**Figure 2**). Overall, 60% of US infant RSVH is among those covered by Medicaid.

RSVH Charges: For White, Black, Native American and Other/Unknown race/ethnicity infants, mean RSVH total charges are higher for Medicaid insurance (**Figure 3**), despite Medicaid reimbursing at lower levels. For Hispanic and Asian/Pacific Islander race/ethnicity infants the opposite is true, charges are higher for infants with other/unknown insurance (**Figure 3**). This results in a greater proportion of the US infant RSVH total charges among all infants except White infants being among infants that pay with Medicaid (**Figure 4**). Overall, 63% of RSVH total charges is among infants that pay with Medicaid (**Figure 4**).

**Figure:**
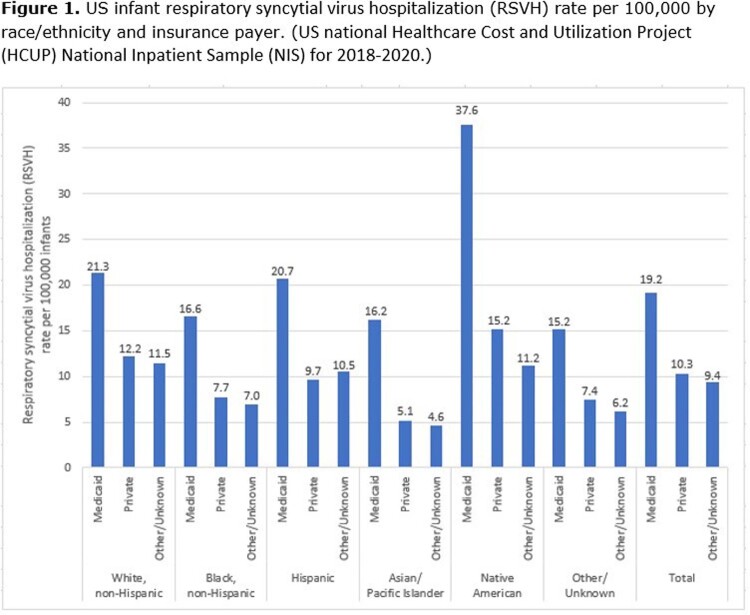

**Figure:**
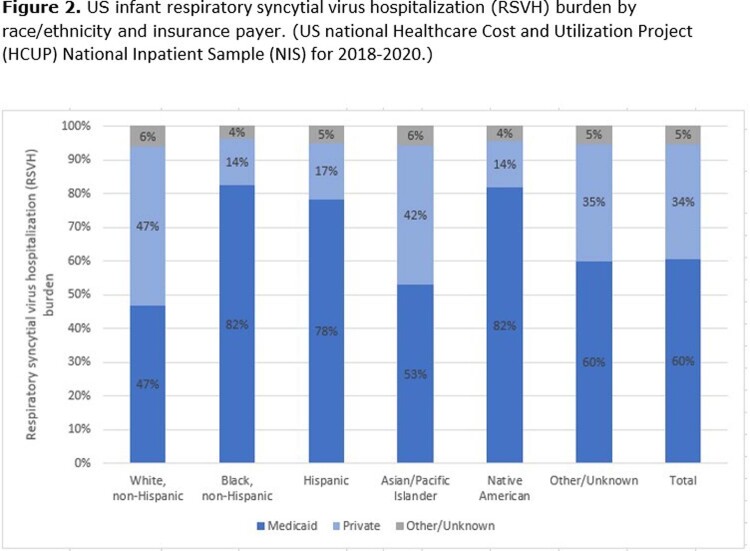

**Figure:**
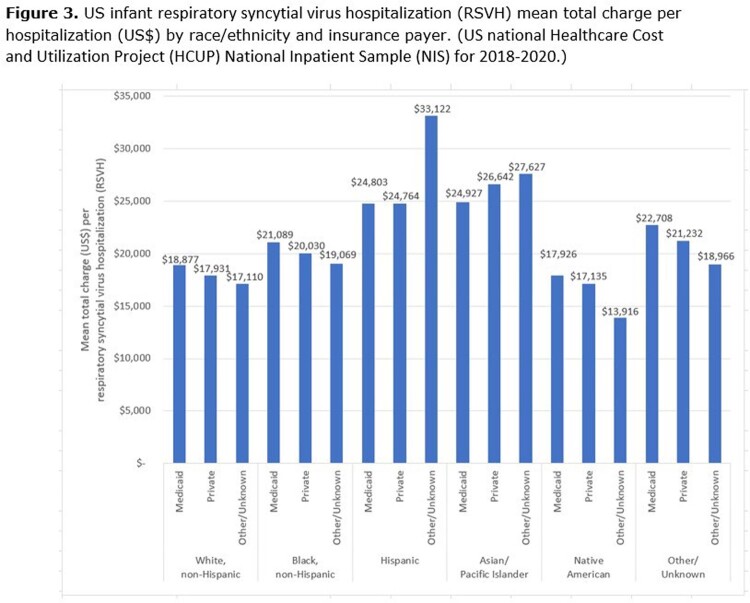

**Conclusion:**

The prevention of RSVH in US infants covered by Medicaid would have a disproportionate benefit for Black, Hispanic, Asian/Pacific Islander, Native American and Other/Unknown race/ethnicity infants and contribute towards economic and race/ethnicity equity.

Because 60% of US infant RSVHs are covered by Medicaid accounting for 63% of RSVH total charges, the prevention of RSVH among infants who paid through Medicaid would also have a substantial impact on overall RSVH burden and related costs.

**Figure:**
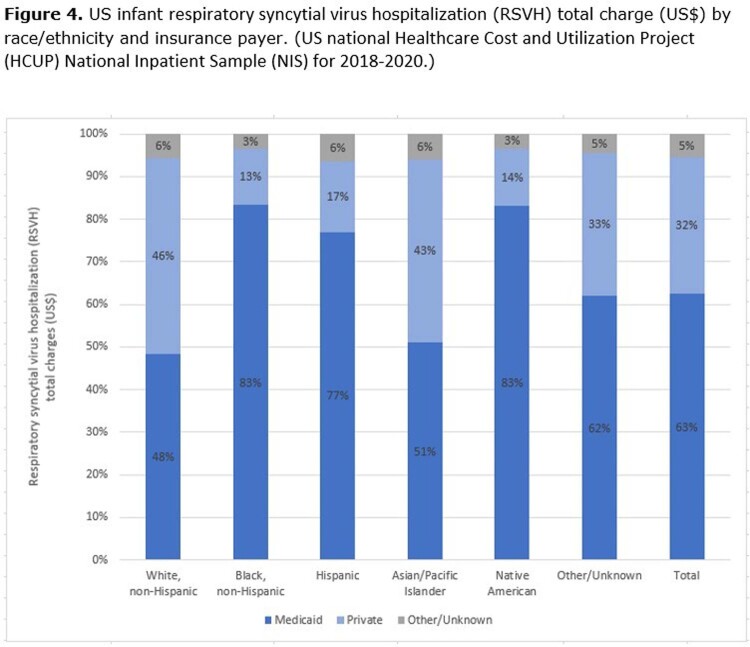

**Disclosures:**

**Christopher B. Nelson, PhD MPH**, Sanofi: employee|Sanofi: employee|Sanofi: Stocks/Bonds|Sanofi: Stocks/Bonds **Xiaohui Jiang, MS**, Sanofi: Grant/Research Support **Mina Suh, MPH, International Health**, AstraZeneca: Grant/Research Support|Sanofi: Grant/Research Support|Sobi: Grant/Research Support **Jon Fryzek, PhD, MPH**, Sanofi: Grant/Research Support

